# The effects of vitamin D levels on physical, mental health, and sleep quality in adults: a comprehensive investigation

**DOI:** 10.3389/fnut.2024.1451037

**Published:** 2024-11-15

**Authors:** Anurag Kumar Singh, Sachin Kumar, Shivang Mishra, Sumit Rajotiya, Sourav Debnath, Preeti Raj, Hemant Bareth, Mahaveer Singh, Deepak Nathiya, Balvir Singh Tomar

**Affiliations:** ^1^Department of Pharmacy Practice, NIMS Institute of Pharmacy, NIMS University Rajasthan, Jaipur, India; ^2^School of Health Science, Faculty of Biology, Medicine and Health, University of Manchester, Manchester, Academic Health Science Center, Manchester, United Kingdom; ^3^Department of Endocrinology, National Institute of Medical Science and Research Hospital, NIMS University Rajasthan, Jaipur, India; ^4^Department of Clinical Studies, Fourth Hospital of Yulin (Xingyuan), Yulin, Shaanxi, China; ^5^Department of Clinical Sciences, Shenmu Hospital, Shenmu, Shaanxi, China; ^6^Institute of Pediatric Gastroenterology and Hepatology, NIMS University Rajasthan, Jaipur, India

**Keywords:** vitamin D, SF-36, ISI, mental health, physical health, sleep

## Abstract

**Background:**

Vitamin D, essential hormone for endocrine, autocrine, and paracrine functions. A billion people are deficient globally which contributing to numerous health issues. This study explores the link between vitamin D levels and sleep quality, impacting mental and physical health in adults.

**Methods:**

This prospective cross-sectional study was conducted at Nims Hospital, Jaipur, involving 484 adults’ participants. Blood samples were collected for serum 25(OH) D measurements. Data were gathered using the SF-36 and ISI questionnaires to assess health and sleep quality.

**Results:**

Higher vitamin D levels were strongly linked to better physical health, including physical function (*r* = 0.642, *p* < 0.001), general health (*r* = 0.560, *p* < 0.001), and PCS score (*r* = 0.441, *p* < 0.001). Vitamin D also positively impacted social functioning (*r* = 0.096, *p* = 0.035) and was negatively related to ISI scores (*r* = −0.112, *p* = 0.014).

**Conclusion:**

The study highlights a strong link between higher vitamin D levels and improved physical and mental health, with significant negative correlation to ISI scores. This underscores the importance of adequate vitamin D for overall well-being. The findings call for urgent measures to address vitamin D deficiency and further research into its health impacts.

## 1 Introduction

Vitamin D, often celebrated as the “sunshine vitamin,” is a pivotal hormone with broad-reaching roles in the body’s endocrine, autocrine, and paracrine systems. This essential nutrient supports bone health, enhances calcium absorption, modulates immune function, and plays a preventive role against a wide range of health conditions, including osteoporosis, autoimmune diseases, and certain cancers (1–3). Despite its importance, about a billion people globally are affected by insufficient vitamin D levels, including 7.4% of Canadians, 5.9% of Americans, and 13% of Europeans (4–6). Deficiency rates are similarly high in countries like Australia, Turkey, and across regions in Africa and South America, indicating a pressing global concern (1, 3, 7, 8). India also reflects this trend, with studies showing deficiency rates between 50% and 94% across diverse population groups (9).

The health implications of vitamin D deficiency are profound. Beyond its foundational role in calcium and phosphorus metabolism, vitamin D insufficiency is linked to an increased risk of chronic illnesses such as osteoporosis, rickets, and serious conditions including breast and colon cancer, cardiovascular disease, hypertension, and diabetes. It has even been associated with neurodegenerative conditions such as Parkinson’s disease, while also impacting mental health by contributing to mood disorders, like depression, which are exacerbated by low vitamin D levels (10). These links became especially relevant during the COVID-19 pandemic, when vitamin D’s immune-modulating properties gained attention for potentially reducing complications related to the virus (11). These extensive connections highlight how vitamin D plays an essential role not only in preventing physical diseases but also in supporting mental well-being, influencing disease susceptibility and overall health across all ages (12–20). In 2019 alone, insufficient vitamin D levels were estimated to affect the mental health of 293 million individuals worldwide (21).

Vitamin D’s influence extends to sleep, an essential aspect of daily functioning and well-being. A deficiency in vitamin D is connected to various sleep disorders, including restless legs syndrome and sleep apnea, which can further exacerbate chronic health conditions through disrupted sleep cycles and diminished sleep quality (22–26). Sleep itself is regulated by a complex interaction of circadian rhythms, neural pathways, and hormonal signals originating in the hypothalamus, which responds to environmental cues like light. Nevertheless, sleep disorders remain underdiagnosed, even within healthcare settings. Research underscores that both excessive sleep and sleep deprivation are associated with heightened risks of diabetes, hypertension, cancers, and increased mortality rates (27).

The relationship between vitamin D insufficiency and sleep disruption has thus emerged as a significant area of study. Vitamin D receptors play a role in producing melatonin, the hormone central to regulating the sleep–wake cycle, supporting mood, and sustaining an optimal quality of life (28–31). Furthermore, geographic and cultural factors, particularly in India, intensify the need to investigate this link. While India enjoys abundant sunlight, limited outdoor exposure, urban lifestyles, and dietary preferences restrict sufficient vitamin D synthesis among large segments of the population. Additionally, factors like dietary restrictions and skin pigmentation can further reduce vitamin D synthesis, elevating deficiency risks. Given the high prevalence of vitamin D insufficiency in India and its potential impact on both physical and mental health, a comprehensive study examining the connection between vitamin D levels and sleep quality is crucial. This investigation aims to explore how these factors jointly influence well-being in adults aged 18 and older, with insights that could improve health outcomes and highlight the importance of vitamin D in maintaining quality of life across diverse lifestyles and geographies.

## 2 Materials and methods

### 2.1 Study design and patient enrollment

A prospective cross-sectional study was conducted at the NIMS hospital, A Tertiary Teaching Care Hospital, A unit of NIMS University Rajasthan, Jaipur, North India for a duration of 8 months (from August 2023 to March 2024). Patients aged 18 years and above visiting the outpatient (OPD) and inpatient (IPD) departments of general medicine without a clinical diagnosis of any bone-related disease and history of bone fractures were enrolled. Whereas, the patients undergoing hormone replacement therapy, diagnosed with HIV, malignant conditions, chronic kidney disease, coronary artery disease, angina, myocardial infarction, history of experiencing a heart attack and drug abuse, consumption of vitamin D supplements, women who were pregnant or breastfeeding, and the people who did not willingly participate or give their consent were excluded. The blood samples of the enrolled participants were collected for serum 25(OH) D measurements in the department of biochemistry and laboratory services at the NIMS hospital. The vitamin D and serum calcium levels were determined as per the standard guidelines of Virtus 5600 (32). Checking serum 25(OH)D levels was done with the Virtus 5600 integrated system [Model number J56001308].

### 2.2 Study recruitment and data collection process

**Figure 1:**
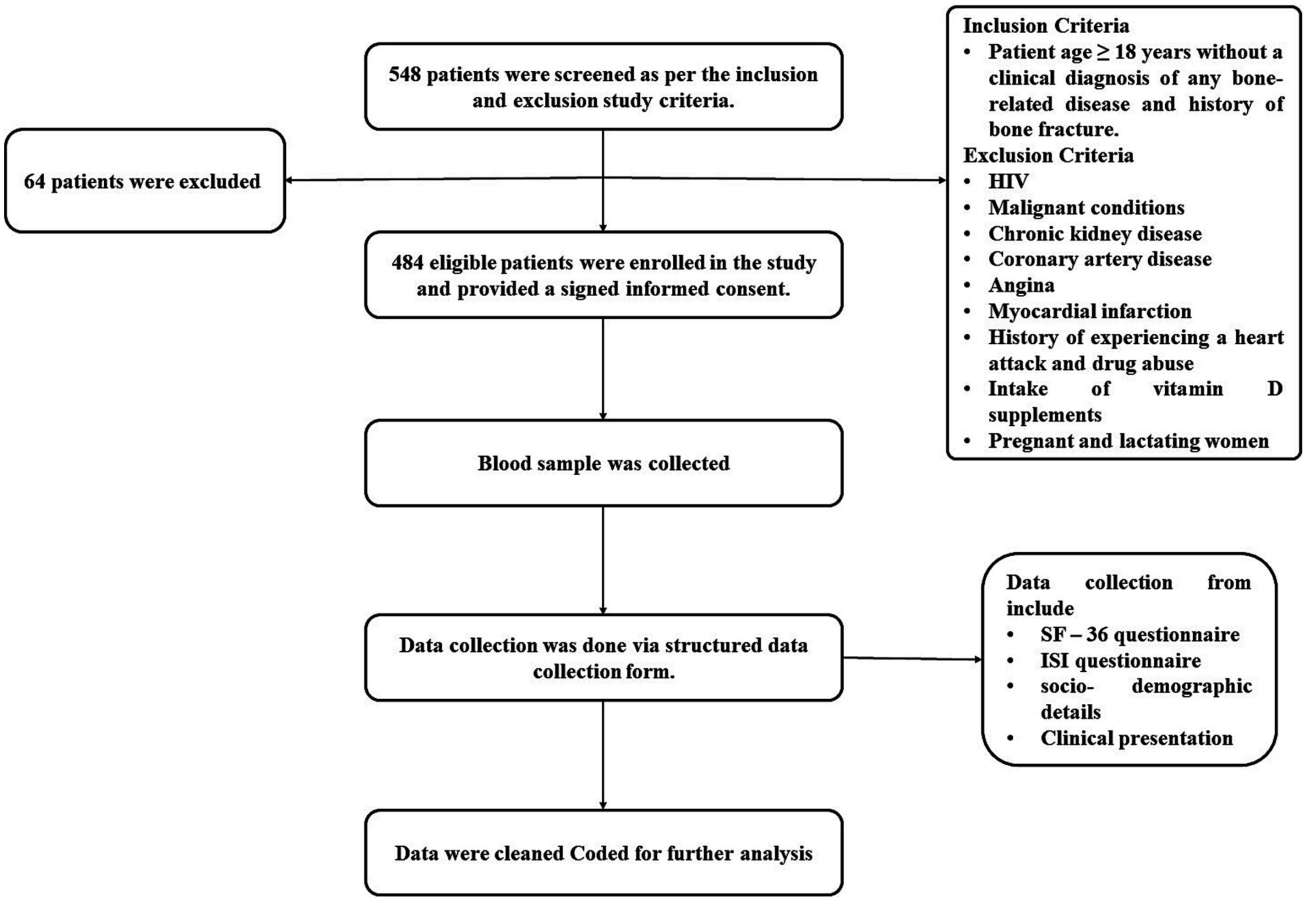
Flowchart illustrating the methodology of the study.

548 patients were screened using the planned inclusion and exclusion criteria. Of these, 64 patients did not meet the inclusion criteria, leaving 484 patients who were eligible to join the study after giving written consent. A structured data collection form with the SF-36 questionnaire, a commonly used tool for evaluating patients’ mental and physical health, was used to collect the data. Physical health and mental health are the two-summary metrics derived from its eight scales. Physical functioning (10 components), role-physical (four items), bodily pain (two items), and overall health make up the physical health summary score (five items). Physical health (four items), social skills (two items), emotional and role-related skills (three items), and mental health make up the mental health measure (five items). These domains evaluate energy levels, social engagement, the impact of emotional issues on daily activities, and overall mental well-being. The RAND Healthcare SF-36 scoring instructions from the RAND Corporation are used to decide the scores. Along with the Insomnia Severity Index (ISI) questionnaire, domain scores range from 0 to 100, with higher scores indicating better health-related quality of life and lower scores indicating worse health. The seven-item ISI questionnaire was used to find out the insomnia’s nature, severity, and effects. Participants were asked about sleep maintenance, sleep onset, sleep dissatisfaction, early morning waking issues, daytime functioning, and anxiety related to sleep difficulties for the past month. Four levels of insomnia severity were found based on the answers: no insomnia (0–7 level), sub-threshold insomnia (8–14 score), moderate insomnia (15–21 score), and severe insomnia (22–28 score) (33–41), encompassing socio-demographic details (such as name, age, gender, address, residential area, marital status, history of smoking, alcohol and tobacco use, milk consumption, and sun exposure), clinical presentation (concerns related to bones, history of bone fractures, comorbidity, current symptoms, and present diagnosis), and all questions from the questionnaires were duly addressed (Figure 1).

### 2.3 Ethics

The research conducted in this study obtained approval from the Institutional Ethics Committee (IEC) at NIMS University Rajasthan. The reference number for this approval is NIMSUR/IEC/2023/677. It is important to note that throughout the entire research process, strict adherence was maintained to the ethical principles delineated in the Declaration of Helsinki, which was established in 1975.

### 2.4 Statistical analysis

The statistical analysis for this study was conducted using two software tools: Excel (version 2019) and IBM SPSS (version 28.0). Power analysis with a power of 0.80 with an alpha level of 0.05, and a confidence interval of 95% was used to reach the required sample size (42). Continuous variables were summarized by calculating the mean and standard deviation, while categorical variables were presented through median, frequency, and percentage measures. To compare categorical variables, the Fisher exact test was employed, whereas for comparing quantities, the *t*-test was utilized. This approach ensured comprehensive and accurate data interpretation in accordance with the study’s objectives.

## 3 Results

A total of 484 participants were enrolled with an average age of 46.60 ± 16.4 years, 43.8% male and 56.2% female. The majority of participants followed a vegetarian diet (84.5%), while 12.7% followed a non-vegetarian diet. There were people from rural areas (59.3% of the participants) and people from cities (40.7%). 25.2% were smokers, with 14.3% being former smokers. Additionally, 19.6% were identified as alcoholics, with 13.4% being former alcoholics. Regarding chewing tobacco, 10.7% were consumers, while 27.1% were former consumers. The average milk consumption was 202.6 ± 133.48 mL/day, while the average sun exposure received was 16.79 ± 8.14 min/day. Based on Body Mass Index (BMI) categorization, 9.03% of participants were classified as underweight, 80.78% as normal weight, 7.64% as overweight, and 2.47% as obese (Table 1).

**Table 1:** Demographic and anthropometric details of the subjects enrolled in the study.

Variables	***N* (%)**
Total subjects (*n*)	**484**
Age (mean ± SD, years)	46.60 ± 16.4
Gender	
Male	212 (43.8%)
Female	272 (56.2%)
Dietary pattern	
Veg	60 (84.5%)
Non-Veg	9 (12.7%)
Area of population	
Rural population	287 (59.3%)
Urban population	197 (40.7%)
Smoking status	
Smoking former	69 (14.3%)
Smoking current	122 (25.2%)
Smoking never	293 (60.5%)
Alcohol status	
Alcohol former	65 (13.4%)
Alcohol current	95 (19.6%)
Alcohol never	324 (66.9%)
Tobacco status	
Tobacco former	131 (27.1%)
Tobacco current	52 (10.7%)
Tobacco never	301 (62.2%)
Body mass index (kg/m^2^)	
Underweight	44 (9.03%)
Normal weight	391 (80.78%)
Overweight	37 (7.64%)
Obese	12 (2.47%)
Milk consumption (mean ± SD, mL)	202.6 ± 133.48
Sun exposure (mean ± SD, min)	16.79 ± 8.14

All the continuous variables are presented in mean ± standard deviation (SD) and categorical variables are presented in number and percentage (%).

### 3.1 Laboratory investigations

There were 70.5% subject with low (≤30 ng/mL), 28.7% normal (30.1–100 ng/mL), and 0.8% high (>100 ng/mL) vitamin D levels with an average of 26.43 ± 15.41 ng/mL. 46.5% subjects were having low (≤8.7 mg/dL), 51.7% normal (8.8–10.7 mg/dL), and 1.9% high (>10.8 mg/dL) serum calcium levels, accounting for an average of 8.72 ± 1.19 mg/dL (Table 2).

**Table 2:** Vitamin D and serum calcium levels of the individuals enrolled in the study.

Variables	N (%)
Vitamin D (ng/mL), Mean ± SD	26.43 ± 15.41
Low 1–30	341 (70.5%)
Normal 30.1–100	139 (28.7%)
High 100.1+	4 (0.8%)
Serum calcium (mg/dL), Mean ± SD	8.72 ± 1.19
Low 0–8.7, *n* (%)	225 (46.5%)
Normal 8.8–10.7	250 (51.7%)
High 10.8+	9 (1.9%)

All the continuous variables are presented in mean ± standard deviation (SD) and categorical variables are presented in number and percentage (%).

### 3.2 Scores of participants for the mental and physical components (SF-36)

For the mental component, the overall score is 27.83 ± 11.36, and it is split into four areas: Vitality, Social Functioning, Role Emotional, and Mental Health. These areas have average scores of 76.15 ± 13.94, 77.81 ± 15.79, 32.57 ± 38.94, and 51.80 ± 11.62, respectively. Physical function, Body Pain, Role Physical and General Health each have an average score of 46.94 ± 15.54, 34.75 ± 26.79, 45.17 ± 14.76, and 44.29 ± 5.17, respectively, on the physical component summary score (52.21 ± 13.83) (Table 3).

**Table 3:** Mental and physical component scores of the individuals enrolled in the study (SF-36).

Variables	Mean ± SD
PCS	52.21 ± 13.83
Physical function	46.94 ± 15.54
Role physical	34.75 ± 26.79
Body pain	45.17 ± 14.76
General health	44.29 ± 5.17
MCS	27.83 ± 11.36
Vitality	76.15 ± 13.94
Social functioning	77.81 ± 15.79
Role emotional	32.57 ± 38.94
Mental health	51.80 ± 11.62

All the continuous variables are presented in mean ± standard deviation (SD).

### 3.3 Insomnia severity index scores of the enrolled participants

The average ISI score of participants was 18.73 ± 6.34, with 3.9% having no clinically significant insomnia, 28.1% with subthreshold insomnia, 14.7% with moderate insomnia, and 53.3% with severe clinical insomnia (Table 4).

**Table 4:** Insomnia severity index of the subjects enrolled in the study.

**Variables**	***N* (%)**
Total (mean ± SD)	18.73 ± 6.34
No clinically significant insomnia	19 (3.9%)
Subthreshold insomnia	136 (28.1%)
Clinical insomnia (moderate severity)	71 (14.7%)
Clinical insomnia (severe)	258 (53.3%)

All the continuous variables are presented in mean ± standard deviation (SD) and categorical variables are presented in number and percentage (%).

### 3.4 Correlation of vitamin D and serum calcium levels with SF-36 (physical, mental health) and ISI score

**Figure 2:**
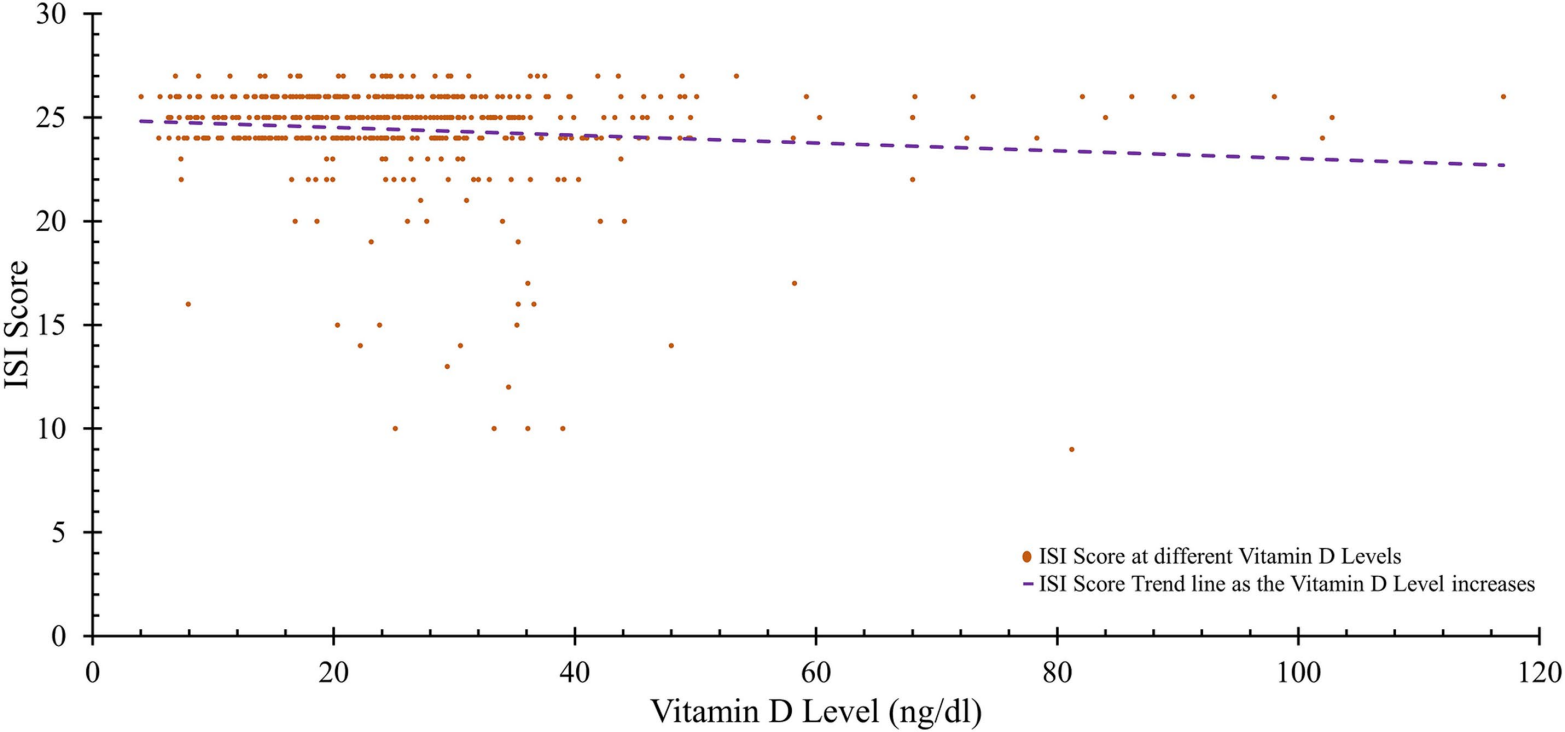
Insomnia severity index of subjects with varying vitamin D levels.

There was a strong link between higher levels of vitamin D and various aspects of physical health, including the physical function score (*r* = 0.642, *p* < 0.001), the general health score (*r* = 0.560, *p* < 0.001), and the physical component summary (PCS) score (*r* = 0.441, *p* < 0.001). Concerning mental health, there was a positive correlation between social functioning and vitamin D levels (*r* = 0.096, *p* = 0.035), while Insomnia Severity Index (ISI) scores were significantly negatively related to vitamin D levels (*r* = −0.112, *p* = 0.014). Additionally, there was a significant positive correlation between serum calcium levels and various aspects of physical health, such as general health (*r* = 0.185, *p* = 0.021) and the PCS score (*r* = 0.018, *p* = 0.047). For mental health, role physical does not show any significant correlation with serum calcium (*r* = 0.050, *p* = 0.276). However, social functioning and role emotional also exhibited no significant correlations with serum calcium (*r* = 0.006, *p* = 0.894 and *r* = −0.051, *p* = 0.267, respectively). ISI scores had a negligible association with serum calcium (*r* = 0.008, *p* < 0.001) (Table 5; Figure 2).

**Table 5:** Correlation of vitamin D and serum calcium levels with SF-36 (Physical, mental health) and ISI score.

Variables	SF-36											ISI
	Physical Health						Mental Health					
	Variables	Physical Function	Role Physical	Body Pain	General Health	PCS	Vitality	Social Functioning	Role Emotional	Mental Health	MCS	
Vitamin D	Pearson correlation coefficient	0.642	0.054	0.011	0.560	0.441	0.018	0.096	0.000	−0.01	0.012	−0.112
	p value*	**0.000**	0.235	0.806	**0.000**	**0.000**	0.690	**0.035**	0.995	0.822	0.800	**0.014**
Serum calcium	Pearson correlation coefficient	0.008	0.050	0.040	0.185	0.018	0.007	0.006	−0.051	−0.045	0.054	0.031
	p value*	**0.000**	0.276	0.377	**0.021**	**0.047**	0.876	0.894	0.267	0.324	0.235	0.495

*The correlation is significant at *p* value < 0.05.

### 3.5 Correlation between SF-36 (physical, mental health) and ISI score

There was a significant negative correlation between ISI score and various aspects of physical health, such as physical function (*r* = −0.193, *p* < 0.001) and general health (*r* = −0.107, *p* = 0.018), with the physical component summary (PCS) score showing a similar trend (*r* = −0.096, *p* = 0.035). Regarding mental health, social functioning was negatively correlated with ISI score (*r* = −0.189, *p* < 0.001) (Table 6).

**Table 6:** Correlation between SF-36 (Physical, mental health) and ISI score.

ISI	SF-36										
	Physical health						Mental health				
	Variables	Physical function	Role physical	Body pain	General health	PCS	Vitality	Social functioning	Role emotional	Mental health	MCS
	Pearson Correlation Coefficient	−0.193	−0.015	−0.026	−0.107	−0.096	0.020	−0.189	0.075	0.034	0.045
	p value*	**0.000**	0.745	0.526	**0.018**	**0.035**	0.665	**0.000**	0.100	0.460	0.326

*The correlation is significant at *p* value < 0.05.

## 4 Discussion

The primary objective of this research was to examine the correlation between vitamin D levels, health-related quality of life (HRQoL) as measured by the SF-36 questionnaire, and the severity of insomnia in adults aged 18 and older without bone-related diseases. Results revealed strong associations between vitamin D levels, several dimensions of HRQoL, and insomnia severity, indicating that vitamin D status significantly affects both physical and mental health outcomes.

Laboratory analyses showed a significant prevalence of vitamin D deficiency, with 70.5% of participants having serum levels below 30 ng/mL, consistent with prior studies emphasizing the high occurrence of vitamin D deficiency in regions with limited sunlight exposure or diets low in vitamin D-rich foods (43). This widespread insufficiency underscores a potential need for interventions to improve vitamin D status as a preventive health measure.

Vitamin D levels were positively correlated with several physical HRQoL dimensions, including physical functioning, general health perception, and overall physical component summary (PCS) scores. Higher vitamin D levels were associated with better physical functioning, fewer limitations in physical role activities, and more positive general health perceptions. This suggests a potential role of vitamin D in reducing physical health limitations and enhancing overall well-being. Additionally, a positive correlation between vitamin D levels and social functioning was observed, indicating that sufficient vitamin D may support better social engagement and interpersonal interactions. While vitamin D’s impact on social functioning is indirect and mediated through its effects on mental health (e.g., depression, social withdrawal), it highlights the multifactorial nature of social behavior, shaped by biological, psychological, and environmental factors (44, 45). Therefore, the complexity of these interactions results in weak but significant correlations, reflecting the nuanced role of vitamin D in social and emotional health (31, 46).

Notably, ISI scores, reflecting insomnia severity, were negatively correlated with vitamin D levels. This suggests that adequate vitamin D levels may help mitigate the severity of insomnia, likely due to the influence of the vitamin D receptor (VDR) on genes related to hormones, neurotransmitters, and circadian rhythm regulation. VDR activation promotes the synthesis of serotonin and melatonin, which are essential for sleep initiation and maintenance. Disrupted VDR signaling can impair these processes, potentially leading to poor sleep quality and disrupted sleep patterns (47). This observation reinforces the relevance of monitoring vitamin D status as part of diagnosing and managing sleep disorders, with implications for both mental and physical health.

Additionally, serum calcium levels were positively associated with physical health aspects within SF-36, particularly in general health and PCS scores, highlighting calcium’s critical role in musculoskeletal and neuromuscular functions. Adequate calcium levels contribute to better muscle function and reduced risks of muscular weakness, as reflected in improved physical function scores. However, serum calcium’s association with ISI scores was minimal, suggesting that calcium levels do not significantly impact sleep-related outcomes. This underscores vitamin D’s unique role in sleep health, distinguishing it from calcium’s primary influence on physical well-being.

Significant correlations between SF-36 physical health parameters—specifically physical functioning and general health—and ISI scores were observed, with lower physical functioning scores associated with higher ISI scores, indicating more severe insomnia in individuals experiencing greater physical limitations. This relationship underscores the interdependent nature of physical impairments and sleep disturbances, as physical limitations may exacerbate the severity of insomnia. Additionally, the significant association between general health perceptions and ISI scores suggests that individuals who perceive poorer overall health also report more severe insomnia, emphasizing how physical and mental health collectively impact sleep quality.

The correlations observed between vitamin D levels, HRQoL, and ISI scores may be partially explained by vitamin D’s involvement in hormonal and neurological regulation. Vitamin D interacts with vitamin D receptors (VDRs), influencing gene expression related to essential hormones and neurotransmitters that maintain physical and mental health. Specifically, VDR activation promotes serotonin and melatonin synthesis, both of which play critical roles in mood regulation and sleep maintenance. Low vitamin D levels can interfere with this regulatory process, potentially disrupting circadian rhythms and aggravating insomnia. Additionally, vitamin D influences melatonin production by binding to receptors in the hypothalamus, which governs circadian alignment; insufficient vitamin D can lead to circadian misalignment and exacerbate sleep disorders, including insomnia and obstructive sleep apnea syndrome (OSAS).

Vitamin D deficiency may also intensify immune and inflammatory responses by elevating pro-inflammatory cytokines, such as IL-6 and TNF-*α*, which are associated with disrupted sleep patterns and poor sleep quality. This inflammatory response may lead to fragmented sleep and worsen airway obstruction, as seen in OSAS (48). Furthermore, vitamin D modulates the body’s hypoxic response via its influence on HIF-1α, potentially mitigating sleep disturbances related to hypoxic episodes. These physiological pathways suggest a complex and multifactorial role of vitamin D in maintaining sleep quality, providing further insights into the observed associations between vitamin D, HRQoL, ISI scores, and overall health (49, 50).

These findings underscore the importance of vitamin D assessments in public health and clinical settings to improve both physical and mental health outcomes. Positive correlations between vitamin D, HRQoL, and insomnia severity suggest that vitamin D status is a modifiable factor with potential to enhance overall quality of life, particularly for populations with high vitamin D deficiency rates. Integrating vitamin D screening and supplementation into healthcare practices may help reduce the burden of sleep disorders and physical impairments, contributing to improved HRQoL and well-being across affected populations. Furthermore, this study contributes to the existing literature by examining vitamin D’s broader impact, not only on physical health but also on sleep quality, mental well-being, and social functioning. This study provides novel insights into vitamin D’s relationship with insomnia severity and social engagement, offering a more comprehensive understanding of vitamin D’s influence on overall HRQoL.

Additionally, the focus on an Indian population with distinct cultural and dietary practices adds valuable perspectives to the global research on vitamin D, highlighting region-specific health interventions and preventive care strategies.

### 4.1 Limitations of the study

The study’s 6-month duration may have limited its ability to fully capture the long-term effects of vitamin D on physical health, mental well-being, and sleep quality. Recruitment challenges due to specific selection criteria and the single-center design further restrict the generalizability of the findings to broader populations or different settings. Additionally, direct measures of sunlight exposure—a key factor in vitamin D synthesis—were not included, potentially affecting the study’s accuracy regarding vitamin D levels and related health outcomes. Future studies would benefit from incorporating direct sunlight measurements to provide a clearer understanding of its impact. Notably, the correlation coefficients for physical and mental health and insomnia were found to be close to or slightly higher than those for insomnia and vitamin D levels, suggesting that the observed effects on sleep quality may arise not only from vitamin D’s direct influence on sleep biochemistry, such as melatonin synthesis, but also through secondary effects on physical and mental health. Expanding future research to longitudinal, multi-site studies with diverse populations could further clarify these relationships, enhancing the generalizability and causal understanding of vitamin D’s role in HRQoL, insomnia severity, and overall health metrics.

## 5 Conclusion

The findings of this study underscore a meaningful connection between higher levels of vitamin D and improved physical and mental well-being, as indicated by better performance on the SF-36 mental health scale and the Physical Component Summary (PCS). Notably, we also observed a significant negative relationship between vitamin D levels and insomnia severity, suggesting that adequate vitamin D may play a vital role in enhancing overall health and well-being. Given these results, there is a pressing need for initiatives aimed at reducing vitamin D deficiency. Additionally, further research is essential to deepen our understanding of the specific mechanisms that link vitamin D to a diverse range of health outcomes.

## Data Availability

The raw data supporting the conclusions of this article will be made available by the authors, without undue reservation.
